# Estimation of the frequency of inherited germline mutations by whole exome sequencing in ethyl nitrosourea-treated and untreated *gpt* delta mice

**DOI:** 10.1186/s41021-016-0035-y

**Published:** 2016-04-01

**Authors:** Kenichi Masumura, Naomi Toyoda-Hokaiwado, Akiko Ukai, Yoichi Gondo, Masamitsu Honma, Takehiko Nohmi

**Affiliations:** Division of Genetics and Mutagenesis, National Institute of Health Sciences, 1-18-1 Kamiyoga, Setagaya-ku, Tokyo, 158-8501 Japan; RIKEN BioResource Center, 3-1-1 Koyadai, Tsukuba, Ibaraki, 305-0074 Japan; Biological Safety Research Center, National Institute of Health Sciences, 1-18-1 Kamiyoga, Setagaya-ku, Tokyo, 158-8501 Japan

**Keywords:** Exome sequencing, Germline mutation, Heritable mutation, Mutation spectrum, Transgenic rodent gene mutation assay

## Abstract

**Background:**

Germline mutations are heritable and may cause health disadvantages in the next generation. To investigate trans-generational mutations, we treated male *gpt* delta mice with *N*-ethyl-*N*-nitrosourea (ENU) (85 mg/kg intraperitoneally, weekly on two occasions). The mice were mated with untreated female mice and offspring were obtained. Whole exome sequencing analyses were performed to identify *de novo* mutations in the offspring.

**Results:**

At 20 weeks after the treatment, the *gpt* mutant frequencies in the sperm of ENU-treated mice were 21-fold higher than those in the untreated control. Liver DNA was extracted from six mice, including the father, mother, and four offspring from each family of the ENU-treated or untreated mice. In total, 12 DNA samples were subjected to whole exome sequencing analyses. We identified *de novo* mutations in the offspring by comparing single nucleotide variations in the parents and offspring. In the ENU-treated group, we detected 148 mutation candidates in four offspring and 123 (82 %) were confirmed as true mutations by Sanger sequencing. In the control group, we detected 12 candidate mutations, of which, three (25 %) were confirmed. The frequency of inherited mutations in the offspring from the ENU-treated family was 184 × 10^−8^ per base, which was 17-fold higher than that in the control family (11 × 10^−8^ per base). The *de novo* mutation spectrum in the next generation exhibited characteristic ENU-induced somatic mutations, such as base substitutions at A:T bp.

**Conclusions:**

These results suggest that direct sequencing analyses can be a useful tool for investigating inherited germline mutations and that the germ cells could be a good endpoint for evaluating germline mutations, which are transmitted to offspring as inherited mutations.

**Electronic supplementary material:**

The online version of this article (doi:10.1186/s41021-016-0035-y) contains supplementary material, which is available to authorized users.

## Background

Gene mutations induced in germ cells may cause heritable changes, which can have adverse health effects on the next generation. Thus, analyses of germ cell mutations and risk evaluations for trans-generational mutagenesis induced by chemicals are important research subjects in genotoxicology [1]. Many genotoxicity and mutagenicity tests employ somatic cells to detect mutagens, which may have carcinogenic potential. Indeed, about 50 mutagens have been analyzed as rodent germ cell mutagens and about 40 of them were positive [2]. Mouse-specific locus tests and mouse heritable translocation tests have been used to detect trans-generational mutations [3, 4]; however, these tests are rarely performed anymore as they need considerably large numbers (hundreds to thousands) of animals. Some genotoxicity/mutagenicity tests are also applicable to germ cells, e.g., transgenic rodent (TGR) gene mutation assays are useful for detecting mutations in both somatic and male germ cells [5]. Recent advances in high-throughput sequencing techniques have extended the use of direct sequencing to mutation analyses. Thus, a combination of TGR mutation assay and next-generation sequencing (NGS) could be a comprehensive approach for analyzing heritable mutations. In this study, we estimated the *N*-ethyl-*N*-nitrosourea (ENU)-induced germline mutation frequency (MF) in *gpt* delta transgenic mice by whole exome sequencing using NGS. ENU is a known germ cell mutagen, and it has been used to generate mutant mice in large-scale chemical mutagenesis projects [6–8]. We treated male mice with ENU and mated them with untreated females at 10 weeks after the treatment. Whole exome sequencing was performed for the parents and offspring of the ENU-treated and control families, and *de novo* germline mutations were detected by comparing single nucleotide variants (SNVs) in the parents and offspring. We also estimated the *gpt* mutant frequency in sperm DNA from the ENU-treated mice. Our results showed that the frequency of inherited mutations was clearly higher in the ENU-treated group than that in the control group. The mutation spectrum determined by NGS indicated the presence of characteristic ENU-induced mutations. Thus, NGS may be a powerful approach for analyzing chemically induced trans-generational mutations, and germ cells could be a good surrogate for trans-generational mutagenesis studies.

## Methods

### Treatment of animal

Male and female *gpt* delta mice (C57BL/6 J background) [9, 10] were obtained from a breeding colony maintained at the National Institute of Health Sciences. The animal treatment employed in this study was approved by the Animal Care and Utilization Committee of the institute. The experimental design was based on a protocol for mouse mutagenesis using ENU [11] and a previously reported RIKEN ENU mutagenesis project [12], with some modifications. Nine-week-old male mice were treated with ENU (85 mg/kg body weight intraperitoneally, weekly on two occasions) (Additional file 1: Figure S1). The male mice were pre-mated with untreated female mice at 6–7 weeks after the last treatment to check for a period of infertility induced by ENU. Exposure to ENU was confirmed by their temporal sterility. Ten weeks after the last treatment, the male mice were mated with untreated female mice and F1 offspring were obtained. Control male mice were treated with phosphate/citrate buffer as vehicle and mated with untreated females without a pre-mating period. Five male mice were used in each group. After the offspring were obtained, male mice were sacrificed at 20 weeks after the last treatment (30 week-old), and their tissues were then collected and stored at −80 °C. The mated females and the offspring were also sacrificed at 30–33 and 5 weeks old, respectively.

### Reporter gene mutation assay

Genomic DNA was extracted from the liver using a RecoverEase DNA Isolation Kit (Agilent Technologies, Santa Clara, CA). Sperm DNA was extracted as described [13] with some modifications. In brief, the cauda epididymis was chopped in 1 mL of phosphate-buffered saline (pH 7.4), filtered, and pelleted by centrifugation. The pellet was re-suspended in 1× saline sodium citrate (SSC) and 0.15 % sodium dodecyl sulfate (SDS). The lysate was centrifuged and the sperm pellet was suspended in 1 mL of 0.2× SSC, 1 % SDS, 1 M 2-mercaptoethanol, and 10 mM EDTA (pH 8.0), before digesting overnight with 0.5 mg/mL proteinase K at 37 °C. DNA was isolated by extracting four times in phenol/chloroform, ethanol precipitation, and re-suspension in TE buffer (pH 8.0). For the *gpt* gene mutation assay, lambda EG10 transgenes were rescued by *in vitro* packaging reactions as phage particles using Transpack Packaging Extract (Agilent Technologies). The *gpt* mutation assay was performed as described previously [14]. In brief, the rescued phages were used to infect *Escherichia coli* strain YG6020, which expressed Cre recombinase to convert the transgene into a plasmid. The infected cells were mixed with molten soft agar and poured onto M9 agar plates containing chloramphenicol (Cm) and 6-thioguanine (6TG). The plates were incubated for 4 days at 37 °C to select colonies that harbored the plasmid carrying the mutated *gpt* gene. Infected cells were also poured onto plates containing Cm without 6TG to determine the number of rescued plasmids. The *gpt* mutant frequencies were calculated by dividing the number of 6TG-resistant colonies by the number of rescued plasmids. The *gpt* mutants obtained from the sperm of ENU-treated mice were sequenced to determine the *gpt* mutations using an ABI3730 sequencer (Applied Biosystems by Life Technologies, Carlsbad, CA) with a sequencing primer gptA2 (5′-TCTCGCGCAACCTATTTTCCC-3′).

### High-throughput DNA sequencing analysis

**Fig. 1:**
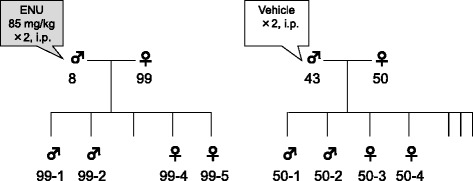
Mouse families used for analyses of whole exome sequencing. For each family, six mice comprising the parents (one male and one female) and four offspring (two males and two females) were used for whole exome sequencing analyses. Each animal ID is presented

One ENU-treated family and one control family were employed in the high-throughput sequencing analyses (Fig. 1), where each family comprised six mice, i.e., parents (male and female) and offspring (two males and two females). Genomic DNA was extracted from the liver using a Wako DNA Extractor WB Kit (Wako, Osaka, Japan). Liver DNA samples from 12 mice were subjected to NGS analyses by Beckman Coulter Genomics (MA, USA). Genomic DNA was fragmented using the Covaris DNA shearing system (Covaris, Inc., MA, USA). The whole mouse exome (49.6 Mb) was captured using a SureSelect Mouse All Exon Kit (Agilent Technologies) and sequenced by a Hiseq2000 (Illumina, CA, USA) with 100-bp paired-ends. Sequenced reads were mapped onto the reference sequence using ELAND (Illumina). The reference sequence was C57BL/6 J mouse genome: NCBI Build 37, mm9 (http://www.ncbi.nlm.nih.gov/projects/genome/assembly/grc/mouse/), and the SureSelect Mouse All Exon Kit was designed using the same reference sequence. For each animal, the SNVs were called based on comparisons with the reference sequence using Samtools.

### Calculation of the frequency of inherited mutations in the offspring of ENU-treated and control fathers

The SNVs were compared in the parents and offspring, and *de novo* mutations were identified as follows. (1) The SNVs transmitted from the parents were excluded. (2) The SNVs found only in one offspring (not found in the other 11 mice) were considered as unique mutations and selected as candidate *de novo* mutations. (3) The read depth, i.e., the number of sequencing reads that covered one nucleotide position, had to exceed 40 in all three mice, i.e., father, mother, and offspring (see the Results). (4) The genotype quality (GQ) scores in NGS had to exceed 20 (=99 % accuracy) in all three mice. The frequency of inherited mutations was calculated as follows. The number of *de novo* mutations was divided by the number of bases in the exome with the same cut-off value, i.e., a read depth ≥ 40 and GQ score ≥ 20 in all three mice. The NGS data were analyzed by Genaris Omics Inc. (Kanagawa, Japan). Confirmation of the mutation candidates by Sanger sequencing was performed by TAKARA BIO Inc. (Shiga, Japan) using an ABI3730 sequencer (Applied Biosystems by Life Technologies) with custom-designed PCR primers for each mutated position in the genome of offspring.

## Results

### ENU treatment and *gpt* mutant frequency

**Fig. 2:**
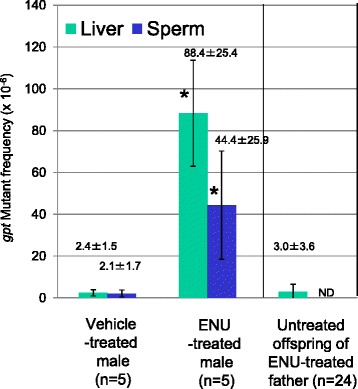
The *gpt* mutant frequencies in the liver and sperm of the ENU-treated mice and their offspring. The *gpt* mutant frequencies are presented with the standard deviations. ND: not determined. Asterisks indicates significant differences vs the vehicle control (*p* < 0.05, Kruskal–Wallis test)

The male mice were treated with ENU and a pre-mating period was enforced to check their temporal infertility due to ENU at weeks 6–7 after the last treatment. No pregnancies occurred during the pre-mating period. At week 10, the mice were mated with untreated females to obtain offspring. In total, 24 F1 mice comprising 17 males and 7 females were obtained from five mated pairs. Tissue samples were collected from the paternal, maternal, and F1 mice. Exposure to ENU was confirmed by the *gpt* mutation assay (Fig. 2). The *gpt* mutant frequency in the liver of ENU-treated mice (88.4 ± 25.4 × 10^−6^) was 37-fold higher than that in the control mice (2.4 ± 1.5 × 10^−6^). In the sperm of ENU-treated mice, the *gpt* mutant frequency (44.4 ± 25.9 × 10^−6^) was 21-fold higher than that of the control mice (2.1 ± 1.7 × 10^−6^). In the offspring of ENU-treated fathers, the *gpt* mutant frequency in the liver (3.0 ± 3.6 × 10^−6^) did not differ significantly from that of the control fathers. One ENU-treated mouse (ID = 8: *gpt* mutant frequency = 74.1 × 10^−6^) and one control mouse (ID = 43: *gpt* mutant frequency = 1.2 × 10^−6^) were selected randomly, and their families with the paired female and offspring were used for the NGS analyses.

### Frequency of inherited mutations in ENU-treated mice

Genomic DNA samples prepared from the livers of one ENU-treated and one vehicle-treated family were subjected to whole exome sequencing (Fig. 1). Initial read data comprising 12–20 gigabases (Gb) were obtained from each animal. The sequenced reads were mapped onto the reference mouse genome sequence, where 54–66 % of the mapped reads were mapped onto the exon region. Thus, over 5 Gb of the sequenced data were mapped onto the 49.6-Mb exon region (approximately 100-fold redundancy). For 70 % of the bases in the exome, the read depth exceeded 30. For each animal, SNVs and small Indels were detected based on comparisons with the reference sequence.

**Fig. 3:**
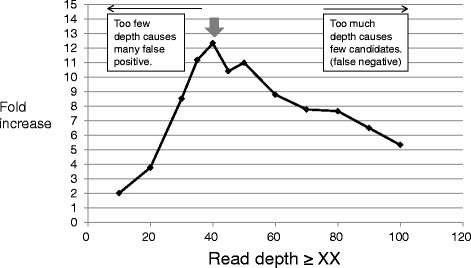
Relative ratio of the number of mutations detected in the offspring of ENU-treated and vehicle-treated fathers. Ratio of the total number of *de novo* candidate mutations in four offspring from the ENU and control group. The thick arrow indicates the peak of the fold increase. When read depth ≥40, the number of mutations in the ENU-treated group was 12-fold higher than that in the control group

The SNVs were compared in the parents and offspring, and *de novo* mutation candidates were scored in the offspring (unique mutations only). The mutation candidates were sorted by using the read depth as a cut-off value. When a higher read depth was set, less mutation candidates were sorted (Additional file 2: Table S1). The ratio of the total number of mutation candidates in the ENU-treated group relative to that of the candidates in the control group was plotted as well as the read depth (Fig. 3). The ratio increased with the read depth and peaked when the read depth ≥ 40, at which it was > 12. This was the most sensitive setting for detecting ENU-induced *de novo* germline mutations under these experimental conditions. Therefore, a read depth ≥ 40 in all three mice (father, mother, and one offspring) was selected as the cut-off value to calculate the frequency of inherited mutations.

**Fig. 4:**
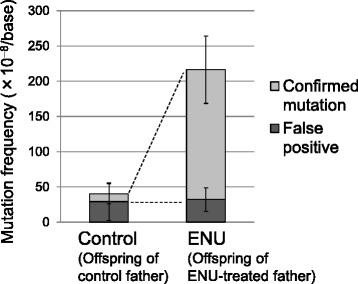
Frequencies of inherited mutations in the offspring of ENU-treated or vehicle-treated fathers. Each bar represents average of 4 offspring with standard deviation. The confirmed mutation frequency was 17-fold higher in the offspring of the ENU-treated father. The frequency of false positives was about 30 × 10^−8^ bases

In the ENU group, 148 candidate mutations were detected from four offsprings, whereas, 12 candidate mutations were detected in the control group (Table 1). Most mutation candidates (157/160) were single base substitutions and three were small indels (two single base deletions and one 2-base deletion) (Additional file 2: Table S2). These candidate mutations were examined by Sanger sequencing. In the ENU group, 83 % (123/148) were confirmed as true mutations, whereas there were many false calls in the control group and only 25 % (3/12) were confirmed. All were detected as heterozygous point mutations in the genomes of the offspring (Additional file 2: Table S2). The MFs of each offspring were calculated by dividing the number of mutations by the number of the bases in exome, which was calculated using the cut-off condition, i.e., read depth ≥40 in all three mice (Additional file 3: Figure S2). The frequency of inherited mutations in the ENU-treated mice was 184 ± 48 × 10^−8^ bases, which was 17-fold higher than that in the control group (11 ± 14 × 10^−8^ bases). In the control group, fewer bases were sorted in the exome compared with the ENU group because the NGS data quality for animal ID 43, which was the father of the control family, was lower compared with the quality for the other mice analyzed. This low quality reduced the number of mutation candidates that exceeded the cut-off value. In this study, the false positive MF was about 30 × 10^−8^ bases in both the ENU and control groups (Fig. 4).

**Table 1:** Frequencies of inherited mutations in the offspring of ENU- or vehicle-treated father

	Offspring ID		Father ID	Mother ID		No. of bases sequenced in exome (depth ≥ 40)	NGS-called mutations (depth ≥ 40)	Confirmed mutations (by Sanger seq)	Confirmed mutation frequency (×10^−8^/base)	Average ± SD
ENU										
	99_1	Male	8	99		18,621,430	35	30	161.1	
	99_2	Male	8	99		13,301,379	42	34	255.6	
	99_4	Female	8	99		18,273,301	35	28	153.2	
	99_5	Female	8	99		18,590,333	36	31	166.8	
					Total	68,786,443	148	123	178.8	184.2 ± 47.9
Control										
	50_1	Male	43	50		6,874,190	2	2	29.1	
	50_2	Male	43	50		6,904,947	4	0	0.0	
	50_3	Female	43	50		6,743,247	2	1	14.8	
	50_4	Female	43	50		6,877,048	4	0	0.0	
					Total	27,399,432	12	3	10.9	11.0 ± 14.0

### Mutation spectra of inherited mutations in ENU-treated mice

The spectrum of inherited mutations in the ENU-treated mice is shown in Table 2. Among 123 confirmed mutations, base substitutions at A:T bps were mainly observed (90/123 = 73.2 %). A:T to G:C transitions occurred in 42.3 % of cases (52/123) and A:T to T:A transversions were observed frequently (33/123 = 26.8 %). The control group had only three confirmed mutations. No mutational hotspots were observed in the ENU-induced mutations (Additional file 2: Table S2). Non-synonymous mutations in coding sequences accounted for 45 % of cases (55/123) and 9 % were synonymous mutations (11/123) (Additional file 2: Table S3). Intronic mutations comprised 19 % of cases (23/123). In two cases, two mutations were identified at neighboring bases in the same animal, where A to G at 27827556 and A to T at 27827557 in chromosome 5 of mouse ID 99_2 may be considered as AA to GT tandem mutations, while T to C at 119834044 and A to T at 119834046 in chromosome 4 of mouse ID 99_5 may be considered as TTA to CTT mutations.

**Table 2:** Mutation spectra in the offspring and somatic tissues of ENU-treated mice

			Control		ENU																	
			NGS (this study)		NGS (this study)		Genome-wide screening^1a^		Transgenic reporter gene mutation assays										Endogenous gene mutation assays			
Target sequences			Whole exome		Whole exome		63 loci (197 Mb)		*gpt* (this study)		*gpt* ^2^		*lacZ* ^3^		*lacZ* ^4^		*lacI* ^5^		*Hprt* (cDNA)^6^		*Hprt* (exon 3)^7^	
Target tissues			Offspring		Offspring		Offspring		Sperm		Bone marow		Liver		Bone marrow		Splenic lymphocytes		Splenic lymphocytes		Splenic lymphocytes	
Mutations			No.	%	No.	%	No.	%	No.	%	No.	%	No.	%	No.	%	No.	%	No.	%	No.	%
Base substitution																						
Transition																						
G:C	to	A:T	1	33.3	21	17.1	32	22.4	11	22.4	17	28.3	6	24.0	7	35.0	13	29.5	4	12.9	2	3.9
A:T	to	G:C	1	33.3	52	42.3	53	37.1	8	16.3	12	20.0	1	4.0	2	10.0	10	22.7	7	22.6	14	27.5
Transversion																						
G:C	to	T:A	0	0.0	12	9.8	13	9.1	7	14.3	9	15.0	6	24.0	4	20.0	4	9.1	1	3.2	1	2.0
G:C	to	C:G	0	0.0	0	0.0	1	0.7	1	2.0	0	0.0	0	0.0	0	0.0	1	2.3	1	3.2	0	0.0
A:T	to	T:A	0	0.0	33	26.8	35	24.5	17	34.7	17	28.3	7	28.0	6	30.0	10	22.7	13	41.9	28	54.9
A:T	to	C:G	0	0.0	5	4.1	8	5.6	4	8.2	3	5.0	3	12.0	0	0.0	4	9.1	5	16.1	6	11.8
Deletion			1	33.3	0	0.0	1	0.7	1	2.0	2	3.3	1	4.0	1	5.0	1	2.3	0	0.0	0	0.0
Insertion			0	0.0	0	0.0	0	0.0	0	0.0	0	0.0	1	4.0	0	0.0	1	2.3	0	0.0	0	0.0
Total			3	100	123	100	143	100	49	100	60	100	25	100	20	100	44	100	31	100	51	100

^1^Male F1 mice by crossing C57BL/6 × DBA/2 or C3H/He [12]

^2^Male BDF1 *gpt* delta mice [10]

^3^Female CD2F1 Muta^TM^ mice [17]

^4^Male CD2F1 Muta^TM^ mice [18]

^5^Male B6C3F1 Big Blue® mice [19]

^6^C57BL/6 mice [30]

^7^Male B6C3F1 mice [19]

^a^Temperature gradient capillary electrophoresis (TGCE) and Sanger sequencing methods

The mutation spectrum of the *gpt* mutations in the sperm of ENU-treated male mice is shown in Table 2, which indicates that A:T to T:A transversions (34.7 %) and G:C to A:T transitions (22.4 %) were mainly observed.

## Discussion

*De novo* mutations in the offspring of the ENU-treated father were identified by whole exome sequencing followed by Sanger sequencing. Theoretically, the minimum dataset required for analyzing *de novo* mutations is a trio (parents and one offspring). However, using such a small sample number may lead to many false positive calls. It is expected that ENU induces point mutations randomly in the genome at a frequency of 1/Mbp [12]. Thus, multiple identical mutations in different individuals could be treated as sequencing errors rather than independent mutations. To minimize the false mutation calls, we counted unique mutations detected in one mouse among the 12 mice used in this study as *de novo* candidate mutations. After simply sorting the unique candidate mutations by read depth ≥ 40 in all three mice (father, mother, and one offspring), the number of candidate mutations in the offspring of the ENU-treated father was over 12 times higher than that in the control. Confirmation by Sanger sequencing indicated that 83 % (123/148) of the ENU-induced *de novo* candidate mutations were true mutations. By contrast, only 25 % (3/12) were confirmed in the control group, and there were many false calls. In this study, false positives mean that Sanger sequencing using the same DNA sample could not confirm the potential NGS mutations (Additional file 2: Table S2). Sanger sequencing may not be able to confirm the mutations in some cases. For example, mismatches or multiple targets of PCR primers may cause failed PCR amplification. In another example, if multiple copies of identical sequence are distributed in the genome, PCR may not preferentially amplify the mutated locus and Sanger sequencing cannot detect a signal of the mutated base. However, there is no perfect explanation for discrepancy between NGS and Sanger sequencing. SNVs detected by NGS may contain some errors even if they have sufficient read depth.

Interestingly, the false positive MFs, i.e., approximately 30 × 10^−8^ bases, was similar in both the control and ENU-treated groups (Fig. 4), which may indicate the background level of false positives under this experimental condition. To reduce false positives, it may be necessary to employ more animals, deeper sequencing, longer target sequences, and to improve the SNV detection methods. However, the false-negative rate was not determined in this study. The candidate mutations were sorted to maximize the sensitivity of the method, i.e., according to the ratio of a number of candidate mutations detected in the ENU and control groups. To avoid underestimating the MF, we calculated the number of nucleotides in the exome, which were sorted by the same condition for mutation detection (read depth ≥40 in all three mice), and the MFs were estimated.

The frequency of confirmed inherited mutations in the ENU-treated father was 184 ± 48 × 10^−8^ bases (Table 1). The RIKEN ENU mutagenesis project estimated the ENU-induced germline MF as 74.9 × 10^−8^ bases according to a genome-wide screening of 197 Mbp of 7472 F1 genomes using temperature gradient capillary electrophoresis (TGCE) and Sanger sequencing [12]. Quwailid et al. performed gene-based screening of 27.4 Mbp of 6000 F1 DNA using denaturing high-performance liquid chromatography (HPLC) and determined a mutation rate of 1 in 1 Mbp [15]. Concepcion et al. analyzed a 9.6 Mb sequence in 510 F1 mice and estimated a mutation rate of 1.04 × 10^−6^ [16]. These ENU mutagenesis studies were performed using a similar dosing design to our study, i.e., two or three weekly intraperitoneal injections with 75–100 mg ENU/kg body weight. Therefore, the ENU-induced MF range estimated by NGS in the present study is comparable to those obtained using other detection methods, such as TGCE, HPLC, and direct sequencing.

The mutation spectrum of the ENU-induced inherited mutations is summarized in Table 2. The characteristic ENU-induced mutations comprised base substitutions at A:T bps, where A:T to G:C (42.3 %) and A:T to T:A (26.8 %) were mainly observed in this study. The mutation spectrum obtained from this NGS-based study appears to be considerably similar to the spectrum produced in another genome-wide analysis using TCGE [12]. However, the predominant type of *gpt* mutation in the sperm of ENU-treated mice was A:T to T:A (34.7 %), followed by G:C to A:T (22.4 %) and A:T to G:C (16.3 %). Transgenic reporter gene assays have demonstrated that the mutation spectra of ENU-induced somatic mutations include base substitutions at both A:T and G:C bps [10, 17–19], where A:T to T:A and G:C to A:T were the predominant mutations in the *lacZ*, *lacI*, and *gpt* genes. This discrepancy may be explained by the different targets used for mutation detection. The TGR mutation assays used reporter genes for *E. coli* or lambda phage integrated in the mouse chromosome, which were not expressed and highly methylated [20, 21]. Whole exome sequencing mainly targets exons that are expressed regions in the genome. ENU reacts directly with DNA and generates a variety of DNA adducts [22, 23], in which *O*^4^-ethylthymine, *O*^2^-ethylthymine, and *O*^6^-ethylguanine are considered to be responsible for causing A:T to G:C, A:T to T:A, and G:C to A:T mutations [24–28]. Transcription-coupled repair may explain some of the differences in the mutation spectra due to the effective repair of specific adducts in the exome sequence, e.g., nucleotide excision repair plays an important role in the repair of guanine adducts [29]. The mutation spectra were also compared with those of an endogenous reporter gene, *Hprt*, in splenic lymphocytes (Table 2). Interestingly, the mutation spectra of *Hprt* showed that A:T base substitutions were most common, as found in the NGS-driven mutation spectrum [19, 30]. However, the major types were A:T to T:A in the *Hprt* locus, but A:T to G:C according to exome sequencing. These differences in the mutation spectra are complex and they do not have simple explanations, e.g., the *Hprt* and transgenic reporter gene assays involve phenotypic selection. Thus, the mutational characteristics were biased by phenotypic changes in the cells caused by the mutated gene products. In addition, although the mutation spectra of *Hprt* were analyzed using cDNA and exon 3, whole exome sequencing includes > 20 % out-of-exon sequences, such as intronic, intergenic, and non-coding regions (Additional file 2: Table S3). The sequence contexts also differ among the genetic regions and organisms. Transgenic reporter genes are bacterial genes and their codon usage differs from that in the mouse. Thus, many factors could have contributed to the differences in the mutation spectra. In principle, genotypic selection might reflect *in vivo* mutagenesis better than phenotypic selection because there is no selection bias. However, it should be noted that NGS also has technical biases in terms of sequencing and mutation detection, such as a preference for point mutations rather than larger indels.

The background control MF (11 ± 14 × 10^−8^ bases) and mutation spectrum are difficult to discuss because of the high standard deviation and the fact only three mutants were confirmed. The germline mutation rate in humans is considered to be about 1 × 10^−8^ per base per generation [31–35]. The control germline and somatic MFs in mice were also estimated as 1 × 10^−8^ per base or less [36, 37]. In the whole exome sequencing, we used a 49.6 Mb target sequence for each mouse. According to the sorting condition employed in this study, i.e., read depth ≥ 40 in all three mice, the passed nucleotide sequences in the exome comprised 13.6–13.9 % in the control group and 26.8 − 37.5 % in the ENU group (Additional file 3: Figure S2). Therefore, the control MF of each animal could have been zero because no mutations were detected. Thus, more control mice and a deeper read depth may be needed to analyze the background MF. Pooling the background data obtained from the exome sequencing of untreated control mice could also be useful for estimating the background MF and standard deviation in laboratory mice.

The *gpt* mutant frequency in the sperm of ENU-treated father (44.4 ± 25.9 × 10^−6^) was 21 times higher than that in the control (2.1 ± 1.7 × 10^−6^). However, the *gpt* mutant frequency in the liver of the ENU-treated father’s offspring was the same as the background level (3.0 ± 3.6 × 10^−6^). If ENU-induced germline *gpt* mutations are transmitted to offspring, then a very high *gpt* mutant frequency could be observed in the F1 mice. It has been reported that there are approximately 40 copies of each transgene per haploid in *gpt* delta mice [38]. If one copy contains the inherited *gpt* mutation, then the expected mutant frequency in the F1 mice would be 1/80 = 12.5 × 10^−3^, which is 1000 times higher than the control level. Based on the 24 F1 mice obtained in this study, we screened 24 × 40 = 960 paternal *gpt* loci. However, we did not observe clonal mutant mice. The *gpt* mutant frequency in the sperm from the ENU-treated mice was 44.4 × 10^−6^, and thus we would require 500 offspring to search for 20000 loci to detect germline mutations at a frequency of 50 × 10^−6^. In fact, Barnett et al. treated male Big Blue^R^ mice with 300 mg ENU/kg body weight and searched for transmitted mutations in 280 offspring, thereby finding four mice with clonal *lacI* mutations in their whole bodies [39]. In contrast, whole exome sequencing could search approximately 50-Mb regions in each offspring. This suggested that high-throughput DNA sequencing analyses could have higher sensitivity to detect inherited mutations using smaller number of animals.

We compare the germline mutagenesis rates measured by NGS and the *gpt* assay in Table 3. The frequency of inherited mutations detected by NGS cannot be compared directly with the *gpt* mutant frequency in sperm, which is based on bacteria-mediated phenotypic selection using a reporter gene, but the increases in the ENU-induced MFs were comparable in both the NGS and *gpt* assay. This suggests that germ cells from the ENU-treated father could be a good endpoint for evaluating germline mutations, which are origins of inherited mutations. It would be interesting to compare the mutational characteristics of germ cell mutations and inherited mutations, but the differences in the mutation spectra obtained by the two methods may reflect different endpoints, e.g., whether the target sequence is expressed or not. It should be noted that the control MF in this whole exome-based study was higher than expected and it had a high standard deviation, and the problem of false positives still needs to be solved. Additional NGS data based on analyses of more families will help to obtain a more accurate estimation of the MF. In particular, more control mice should be analyzed to estimate the background MF. By using a genetically-closed colony of laboratory animals, the sequence data could be pooled from independent experiments. If the sequencing cost decreases, whole genome sequencing will also be a better solution than whole exome sequencing. In addition, genome-wide instability including copy number and structural variations could also be important targets, as well as point mutations. The detection of *de novo* mutations by NGS does not need specific transgenic animals with reporter genes and it can be applied to any organism. Thus, this could be a powerful tool for investigating inherited mutations and evaluating the mutagenic effects of chemicals in the next generation in human populations.

**Table 3:** Summary of the germlime mutagenicity estimated by NGS and TGR mutation assay

	NGS study	TGR mutation assay
	(Confirmed by Sanger seq.)	
Background MF	11 ± 14 × 10^−8^/base^a^	2.1 ± 1.7 × 10^−6^/reporter gene^b^
ENU-induced MF	184 ± 48 × 10^−8^/base^a^	44.4 ± 25.9 × 10^−6^/reporter gene^b^
(Fold increase)	(17-fold)	(21-fold)
Target sequence	Whole exome	Neutral transgene
	(49.6 Mb)	(*gpt*: 456 bps)
Method of detection	Direct sequencing	Bacteria-mediated phenotypic selection
Source of DNA	Liver of offspring	Sperm of treated father
	(Inherited mutation)	(Germ cell mutation)

^a^Independent mutation frequency. Unit = 1. (Each unit contains father, mother and 4 F1s.)

^b^ *gpt* mutant frequency. N = 5

## Conclusions

In this study, we analyzed ENU-induced trans-generational mutations by NGS with Sanger sequencing. ENU-treated and control families of *gpt* delta mice were subjected to whole exome sequencing analyses and we detected *de novo* mutations in the offspring. The frequency of inherited mutations in the offspring of the ENU-treated family was 17 times higher than that in the control family, and it was comparable to the *gpt* mutant frequency in the sperm of the ENU-treated mice. The ENU-induced inherited mutation spectrum determined by NGS identified characteristic base substitutions, such as A:T to G:C and A:T to T:A. These results suggest that direct sequencing analyses using NGS could be a powerful tool for investigating inherited germline mutations and for evaluating the genotoxic effects of mutagens in the next generation. In addition, germ cells could also be a good endpoint for evaluating germline mutations.

## Additional files

Additional file 1: Figure S1. Design of the animal experiment. Paternal (P), maternal (M), and offspring (F1) mice are shown. (PPTX 79 kb) Additional file 2:
**Table S1.** Number of the mutation candidates in the offspring of ENU-treated and vehicle-treated fathers. **Table S2.** List of the NGS-detected de novo mutations in the offspring of ENU-treated and control father. **Table S3.** Types of the confirmed de novo mutations detected by whole exome sequencing. (XLSX 40 kb) Additional file 3: Figure S2. Percentage of nucleotides in the exome sorted according to the same condition for mutation detection in each of the offspring. Sorting conditions: read depth ≥40 and GQ score ≥20 for each of the three mice (father, mother, and one offspring). (PPTX 187 kb)

## Abbreviations

6TG: 6-thioguanine
Cm: chloramphenicol
ENU: *N*-ethyl-*N*-nitrosourea
Gb: Gigabase
GQ: genotype quality
HPLC: high-performance liquid chromatography
MF: mutation frequency
NGS: next-generation sequencing
SDS: sodium dodecyl sulfate
SNV: single nucleotide variant
SSC: saline sodium citrate
TGCE: temperature gradient capillary electrophoresis
TGR: transgenic rodent
