# Supplementary figures and images for: Estimation of the frequency of inherited germline mutations by whole exome sequencing in ethyl nitrosourea-treated and untreated gpt delta mice
Source: Genes Environ. 2016 Apr 1;38:10. doi: 10.1186/s41021-016-0035-y (PMC4918133; doi:10.1186/s41021-016-0035-y)

## Slide 1
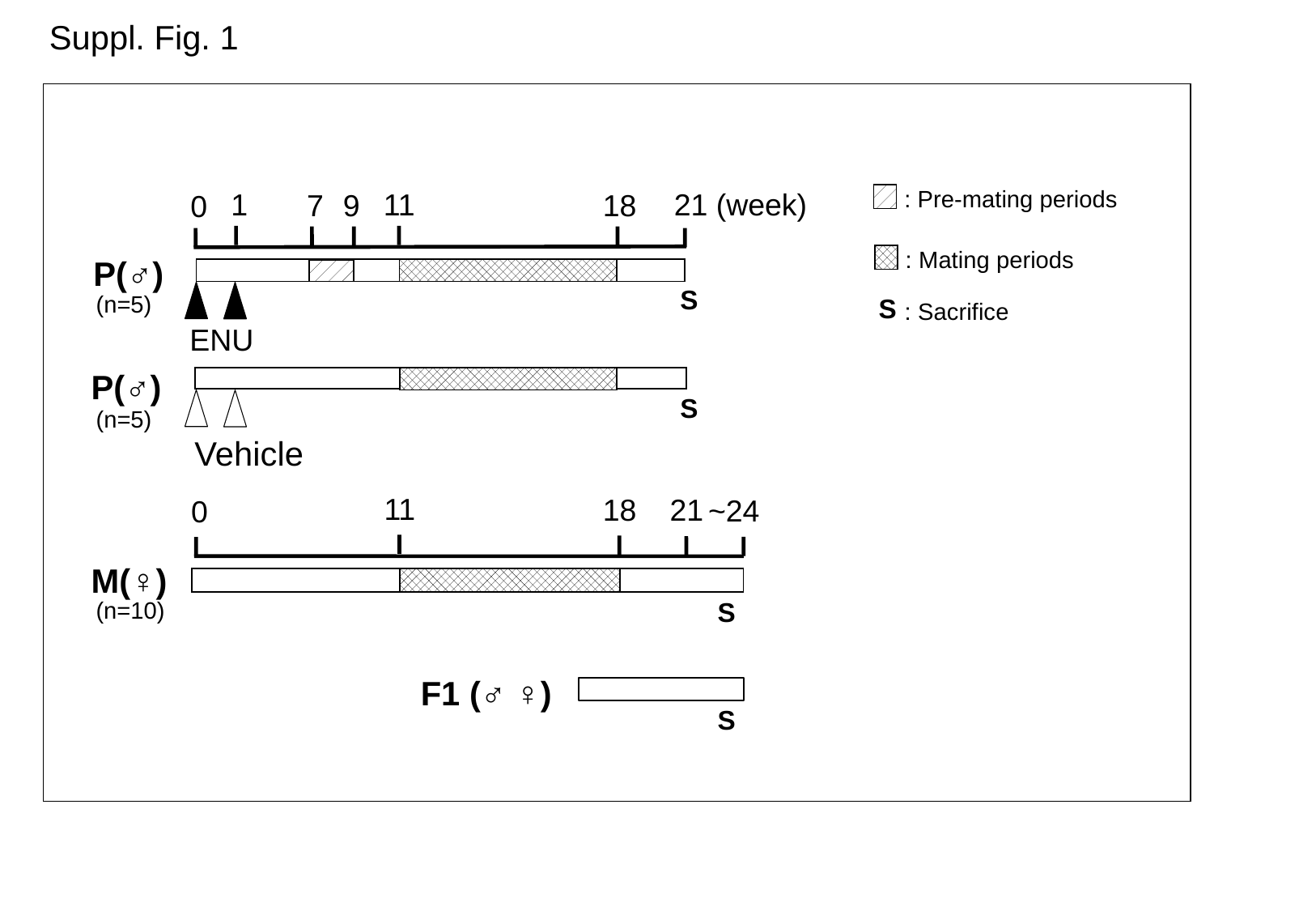

Suppl. Fig. 1
: Pre-mating periods
1
11
21 (week)
7
9
18
0
: Mating periods
P(♂)
S
(n=5)
S
: Sacrifice
ENU
P(♂)
S
(n=5)
Vehicle
11
18
21
~24
0
M(♀)
S
(n=10)
F1 (♂ ♀)
S

Supplement: Additional file 1: Figure S1. — Design of the animal experiment. Paternal (P), maternal (M), and offspring (F1) mice are shown. (PPTX 79 kb) [file 41021_2016_35_MOESM1_ESM.pptx]

## Slide 1
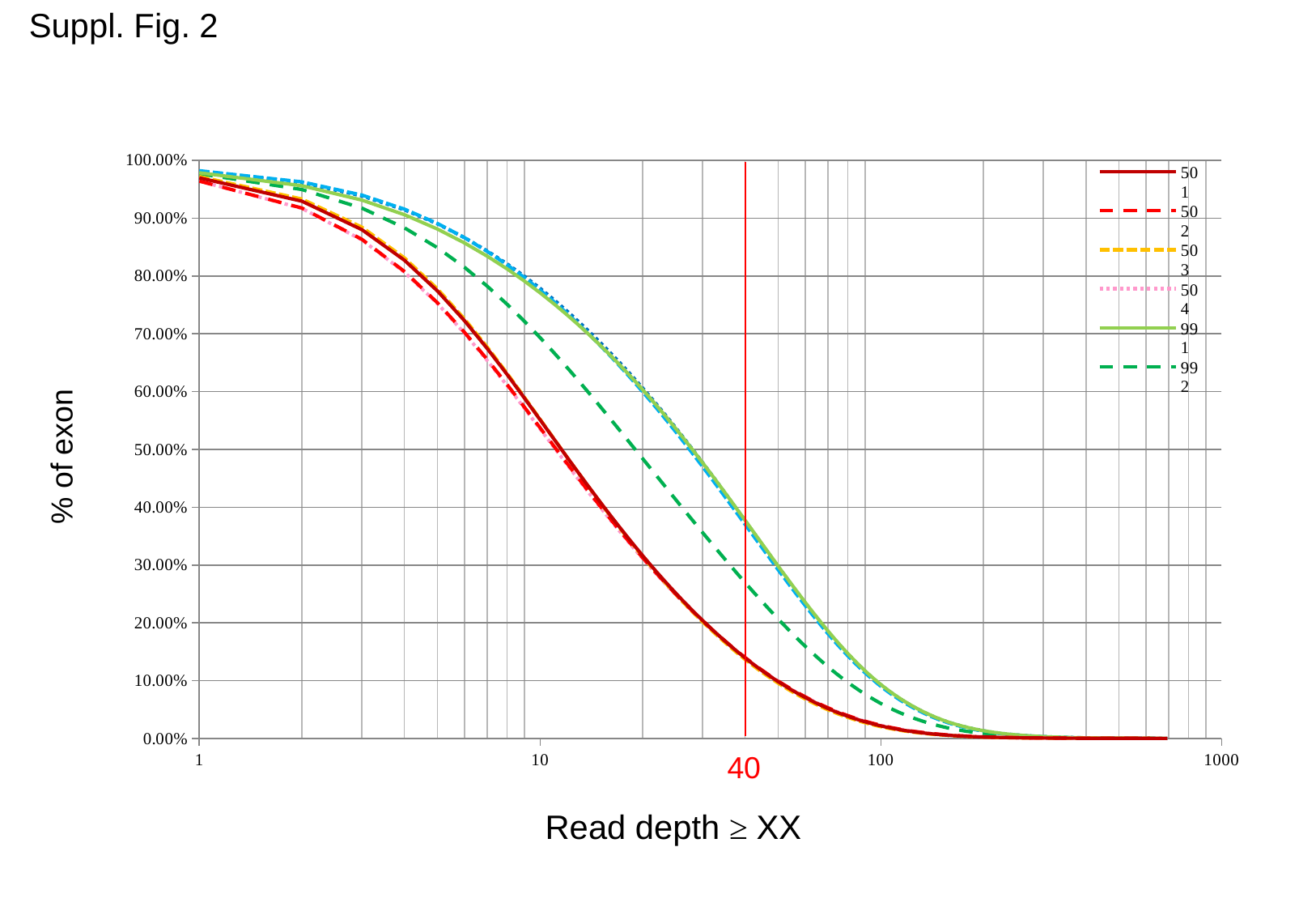

Suppl. Fig. 2
### Chart
| Category | | | | | | | | |
|---|---|---|---|---|---|---|---|---|% of exon
40
Read depth ≥ XX

Supplement: Additional file 3: Figure S2. — Percentage of nucleotides in the exome sorted according to the same condition for mutation detection in each of the offspring. Sorting conditions: read depth ≥40 and GQ score ≥20 for each of the three mice (father, mother, and one offspring). (PPTX 187 kb) [file 41021_2016_35_MOESM3_ESM.pptx]
